# Pilot study: an acute bout of high intensity interval exercise increases 12.5 h GH secretion

**DOI:** 10.14814/phy2.13563

**Published:** 2018-01-22

**Authors:** Sarah E. Deemer, Todd J. Castleberry, Chris Irvine, Daniel E. Newmire, Michael Oldham, George A. King, Vic Ben‐Ezra, Brian A. Irving, Kyle D. Biggerstaff

**Affiliations:** ^1^ Department of Kinesiology Texas Woman's University Denton Texas; ^2^ Department of Kinesiology University of Texas at El Paso El Paso Texas; ^3^ School of Kinesiology Louisiana State University Baton Rouge Louisiana

**Keywords:** Deconvolution analysis, growth hormone, high‐intensity interval exercise, HIIT

## Abstract

The purpose of this study was to test the hypothesis that high‐intensity interval exercise (HIE) significantly increases growth hormone (GH) secretion to a greater extent than moderate‐intensity continuous exercise (MOD) in young women. Five young, sedentary women (mean ± SD; age: 22.6±1.3 years; BMI: 27.4±3.1 kg/m^2^) were tested during the early follicular phase of their menstrual cycle on three occasions. For each visit, participants reported to the laboratory at 1700 h, exercised from 1730–1800 h, and remained in the laboratory until 0700 h the following morning. The exercise component consisted of either 30‐min of moderate‐intensity continuous cycling at 50% of measured peak power (MOD), four 30‐s “all‐out” sprints with 4.5 min of active recovery (HIE), or a time‐matched sedentary control using a randomized, cross‐over design. The overnight GH secretory profile of each trial was determined from 10‐min sampling of venous blood from 1730–0600 h, using deconvolution analysis. Deconvolution GH parameters were log transformed prior to statistical analyses. Calculated GH AUC (0–120 min) was significantly greater in HIE than CON (*P* = 0.04), but HIE was not different from MOD. Total GH secretory rate (ng/mL/12.5 h) was significantly greater in the HIE than the CON (*P* = 0.05), but MOD was not different from CON or HIE. Nocturnal GH secretion (ng/mL/7.5 h) was not different between the three trials. For these women, in this pilot study, a single bout of HIE was sufficient to increase 12.5 h pulsatile GH secretion. It remains to be determined if regular HIE may contribute to increased daily GH secretion.

## Introduction

Regular physical activity supports weight loss, prevents weight gain, and prevents weight regain after weight loss (Donnelly et al. 2009). Consistent with current physical activity recommendations (~45–5% VO_2max_ for 150–300 min/week) (Donnelly et al. 2009; American College of Sports Medicine, 2014), most evidence‐based weight loss programs center around 30–60 min of moderate‐intensity continuous exercise on most days of the week. However, given reports indicating a “lack of time” as a critical barrier to regular exercise (Trost et al. 2002; Schutzer and Graves 2004), high‐intensity interval exercise (HIE) may be a time‐efficient and effective exercise alternative for potentially reducing body mass and fat mass in overweight/obese individuals (Gibala and McGee 2008; Gillen and Gibala 2014).

High‐intensity interval training (HIIT) is generally defined as a bout of exercise performed at an “all‐out” (>90% VO_2max_) effort, followed by a recovery phase of rest or low‐intensity exercise (Gibala and McGee 2008). While HIIT‐induced aerobic and skeletal muscle adaptations have been reported in as little as 2 weeks (Irving et al. 2011; Little et al. 2011), HIIT is also an effective modality for inducing fat loss involving a significantly less time commitment while achieving similar (and sometimes better) health outcomes as traditional endurance training programs (Tremblay et al. 1994; Trapp et al. 2008; Heydari et al. 2012; Gillen et al. 2013).

The mechanisms responsible for changes in body mass and fat mass with HIE are undetermined, but may include changes in adipose tissue lipolysis through catecholamine and lipolytic hormone responses. Growth hormone (GH) responses to traditional endurance training or strength training have been widely documented, yet the effect of HIE on GH concentrations has not been extensively studied. A single‐30‐sec bout of maximal exercise on a treadmill increased GH concentrations 10‐fold above baseline at 1‐h postexercise (Nevill et al. 1996). Similarly, seven 1‐min bouts of intermittent cycling (absolute intensity of 285 W) in young men significantly elevated GH concentrations above those observed during continuous cycling for 20‐min at 100 W (Vanhelder et al. 1984). Among healthy men, GH concentrations remained elevated above baseline for 3‐h postexercise following a 6‐sec (~217%) or 30‐sec (~530%) all‐out sprint on a cycle ergometer (Stokes et al. 2002). However, the above studies only measured GH concentrations up to a few hours postexercise; the effect a HIE bout on 12 h GH secretion, particularly overnight, when GH pulses are greatest is unclear.

Much of the existing literature regarding GH secretion following exercise has been done in young, healthy males. Given the potent lipolytic effect of GH, and knowing that the magnitude of exercise‐induced GH release is intensity dependent, the effect of HIE on GH secretion may have significant implications for treatment of overweight and obesity in young women. The purpose of this pilot study was to examine the effect of an acute bout of high intensity interval exercise (HIE) compared to moderate‐intensity exercise (MOD) or no exercise (control, CON) on 12.5‐h GH secretion and resting fat oxidation in sedentary young women.

## Materials and Methods

### Participants

Five young sedentary weight stable women (age 21–25 years; BMI 23.0–30.8 kg/m^2^; ∆body mass ± 2 kg for previous 6 months), participated in this study. Sedentary was defined as less than 3 days/week of moderate‐intensity [46–64% $\dot{V}O_{2\max}$] or greater physical activity (American College of Sports Medicine, 2014) with less than 30 min per exercise session for the previous 3 months. Exclusionary criteria for this study were: any known or diagnosed renal, hepatic, hematologic, metabolic, or cardiovascular disorder; current or recent smoking; alcohol consumption (>1 drink per day); and/or regular physical activity (≥3 days/week of moderate‐intensity activity). Further, women who were taking birth control (other than oral contraceptives, *n* = 2), hormone replacement therapy, or recombinant hGH were excluded. Women with diagnosed anemia, or who have irregular menstrual cycles, as well as women who had a history of extreme dietary patterns (such as eating disorder) were excluded from this study. All participants provided IRB‐approved informed consent prior to participation.

### Study design

This experiment was a randomized‐controlled crossover design. Each participant completed a descriptive data collection day (Day 1). All overnight trials (CON, MOD, and HIE) took place when women were in the early follicular phase of their menstrual cycle (i.e., within 2–8 days after onset of menstrual bleeding). The order of these trials (CON, MOD, and HIE) was randomized and separated by at least one normal menstrual cycle (27–35 days) and each participant completed all three trials.

On Day 1, following completion of the informed consent and other forms, blood was collected from an antecubital vein to verify that hepatic, renal, metabolic, and hematologic function were normal and for measurement of lipid profile. Participants’ anthropometric measurements (height, weight, and waist circumference) were taken and they completed a dual‐energy X‐ray absorptiometry (DXA) scan for measurement of body composition and distribution of body fat. Lastly, participants completed an incremental maximal oxygen uptake ($\dot{V}O_{2\max}$) test on an electronically braked cycle ergometer (Velotron; RacerMate, Seattle, WA).

For the trial days (CON, MOD, or HIE), participants were asked to return to the laboratory following a day of rest (no structured exercise) at 1700 h. Beginning at 1730 h participants would: (1) rest quietly in a chair for 30 min (CON); (2) complete 30 min of cycling at 50% of maximum workload achieved during the $\dot{V}O_{2\max}$ test (MOD), or (3) complete 4 Wingate tests on the cycle ergometer (HIE) during a 30‐min time period. Participants were then able to relax in the laboratory until approximately 0700‐h the next morning. During this time (1730–0600 h), blood samples were drawn in 10‐min intervals for assessment of pulsatile GH secretion. From 0630 to 0700‐h, oxygen consumption ($\dot{V}O_{2}$), carbon dioxide production ($\dot{V}{\text{CO}}_{2}$), and nonprotein respiratory quotient (NPRQ) were measured by indirect calorimetry (True One 2400 Metabolic System; ParvoMedics, Sandy, UT). Participants were then asked to return to complete the other trial days when they were again in the early follicular phase of their menstrual cycle.

### Cardiovascular fitness

Cardiovascular fitness ($\dot{V}O_{2\max}$) was determined using a ramp cycling protocol (Little et al. 2010) on an electronically braked cycle ergometer. Thirty‐second averages of expired gasses ($\dot{V}O_{2}$ and $\dot{V}{\text{CO}}_{2}$) and expired ventilation ($\dot{V_{E}}$) were continuously collected by indirect calorimetry. Heart rate was monitored during the test by 12‐lead electrocardiogram (Quinton Q‐Stress; Cardiac Science, Milwaukee, WI).

Briefly, the participant started pedaling at 50 W at a comfortable cadence (minimum of 50 rpm) for 60 sec as a warm‐up. At the start of Minute 2, the load increased by 1 W every 2 sec until the participant reached volitional exhaustion or their pedal cadence dropped below 40 rpm. A successful test ($\dot{V}O_{2\max}$) was determined by achievement of a plateau in $\dot{V}O_{2}$ with increasing work (primary criteria); or if a plateau was not reached, participants needed to achieve the following: (1) heart rate within 10 bpm of age‐predicted HR_max_; (2) RER > 1.10; and (3) blood lactate concentration > 8.0 mmol/L measured by capillary finger‐stick 2 min after the cessation of exercise. Participants were given verbal encouragement throughout the exercise test.

### Trial days

For all trial days, participants were asked to report to the lab at 1700‐h and at least 3‐h postprandial. Participants selected a dinner from the Icon Meals website (Icon Meals, Frisco, TX), and they ate the same meal during each trial at 1930 h (mean ± SD: 30 ± 11% CHO; 18 ± 7% FAT; 52 ± 12% PRO; 397 ± 118 kcals).

#### Control day

For the CON trial, participants sat quietly from 1730–1800 h. Participants were allowed to watch movies, read, or do homework during this time period. At 0‐min, the end of the 30‐min period, 60‐min, 90‐min, and 120‐min, a 25 *μ*L sample of venous blood was taken to measure lactate concentration (YSI 2300; Yellow Springs, OH).

#### Moderate intensity steady‐state exercise

For the MOD trial, beginning at 1730 h, participants cycled at ~50% of maximum power (range: 75–90 W) continuously for 30 min. Actual wattage was decreased if necessary to ensure the participants could complete the full 30 min of exercise. During the last 5‐min of the exercise period, indirect calorimetry was used to determine $\dot{V}O_{2}$, $\dot{V}{\text{CO}}_{2}$, and RER steady‐state values. Heart rate was monitored during exercise using a Polar^®^ heart rate monitor (Polar; Lake Success, NY) that was fitted around the chest. At 0‐min, 5‐min time points throughout the MOD trial, 30‐min (end of exercise), 32‐min, 34‐min, 36‐min, 38‐min, 40‐min, 45‐min, 50‐min, 60‐min, 90‐min, and 120‐min, a 25 *μ*L sample of venous blood was taken to measure lactate. Activity energy expenditure during MOD was calculated using the following equation:

kj=average power (W)×duration (h)×3.6ksec/h

To convert kilojoules to energy expenditure (kcal), we multiplied by 4.184 kcal/kj and by 25% (assumed efficiency of human work).

kcal=kj×4.184kcal/kj×0.25

#### High‐intensity interval exercise

For the High‐intensity interval exercise (HIE) trial, beginning at 1730 h, each participant was asked to complete four, 30‐sec sprints on an electronically braked cycle ergometer following a light warm‐up (5 min at 50 W). The resistance set for the 30‐sec sprint was equal to 6.5% of the participant's body mass for a total high‐intensity exercise time of 2 min. The recovery interval between Wingate tests was 4.5 min, during which the participant was asked to cycle at a low cadence (30–40 rpm) and a light resistance (30 W) to reduce venous pooling in the lower extremities and minimize feelings of nausea or light headedness. At the end of the fourth sprint, participants continued to cycle at 30 W at an easy cadence (30–40 rpm) for 4.5 min of recovery. The total exercise time during the HIE protocol was 30 min (including warm‐up and recovery intervals). Heart rate was monitored during exercise using a Polar^®^ heart rate monitor that was fitted around the chest. Blood samples for lactate analysis were taken at the same time points as MOD and activity energy expenditure for HIE was calculated using the same equations as for MOD. The Velotron Wingate software provided total kilojoules for each sprint.

### Resting substrate utilization

Resting substrate utilization was measured by indirect calorimetry the following morning using the ventilated hood technique as described by the American Dietetic Association (Compher et al. 2006). Briefly, upon waking, and after voiding, participants were placed in a semi‐recumbent position and asked to lie quietly while a rigid, clear, plastic canopy was placed over their head and sealed for the collection of $\dot{V}O_{2}$ and $\dot{V}{\text{CO}}_{2}$ for 30 min. The first 10 min of data was discarded and during the last 20 min, a 10‐min steady‐state (<10% coefficient of variation in $\dot{V}O_{2}$ and $\dot{V}{\text{CO}}_{2}$) was used for calculation of NPRQ after completion of the test. Throughout the overnight trial period, the participant's urine was collected for determination of urinary nitrogen (uN_2_, Stanbio, Boerne, TX) and calculation of NPRQ (Simonson and DeFronzo 1990).

### Blood collection and analysis

Upon arrival to the laboratory at 1700 h, an indwelling catheter was inserted into a forearm vein and attached to a saline drip to prevent clotting. Prior to beginning a trial at 1730 h (CON, MOD, or HIE), a baseline blood sample was taken (at approximately 1720 h) for measurement of estradiol (E_2_), growth hormone (GH), nonesterified fatty acids (NEFA), and lactate. For each lactate time point, approximately 1 mL of blood was placed in a microcentrifuge tube containing a lysing agent for preservation of blood lactate (YSI 2315 Lactate Preservative Kit) and immediately vortexed. Starting at 1730 h, 3‐mL serum blood samples were drawn at 10‐min intervals for measurement of pulsatile GH until 0600 h the following morning (12.5 h). Saline was infused for replacement of fluid lost during blood sampling. After the first hour, NEFA samples were analyzed only at 30‐min time points during the overnight trial.

Serum blood samples were allowed to clot for 10–30 min and then centrifuged at 3000 rpm (4 ± 2°C) for 10 min. Serum aliquots were separated into prelabeled cryule vials and frozen at −80°C until analyzed for hormone concentrations using commercially available ELISA kits. All samples from a participant were analyzed together to eliminate any interassay variability.

#### Human growth hormone ELISA kit (LDN, Germany)

Approximately, 100 *μ*L of serum was needed for duplicate determination of hGH with this ELISA kit. The sensitivity of this assay has been determined to be 0.2 ng·mL^−1^. Samples collected in the present study with a concentrations <0.2 ng·mL^−1^ were assigned a value of 0.2 ng/mL for statistical analysis. The interassay %CV for all GH plates (*n* = 31) was 15% for the low control and 16% for the high control. Since we were not able to get all plates from the same lot, inter‐assay variability was reanalyzed by each individual kit lot – lot #160575: low control 13% and high control 11% (*n* = 13 plates); lot#161218: low control 3% and high control 4% (*n* = 9 plates); and lot#161122: low control 4% and high control 4% (*n* = 9 plates).

### Pulsatile growth hormone secretion

Pulsatile growth hormone secretion was estimated by deconvolution analysis (Veldhuis and Johnson 1988) using the AutoDecon Software (Johnson et al. 2009). Pulse parameters compared between the three trials were: mean GH concentration (ng·mL^−1^), total pulsatile secretion (ng·mL^−1^·12.5 h^−1^), number of detected pulses, interpulse interval (min), pulse height (ng), and pulse area (ng·nL^−1^·min^−1^). The initial half‐life and secretion standard deviations estimates were set to 10 min and 13 min, respectively.

### Statistical analysis

All results are reported as mean ± standard deviation. The dependent variables: GH AUC, NEFA AUC, estradiol, and NPRQ were analyzed using a repeated measures ANOVA with *α* = 0.05. Growth hormone and lactate responses for the first 120 min were analyzed by a repeated measures factorial ANOVA (condition × time) with a *P* < 0.05 indicating statistical significance. Due to a large amount of variability in GH secretion among individuals, the deconvolution parameters were log‐transformed for analysis. A repeated measures ANOVA was used to determine the effect of exercise intensity (CON, MOD, HIE) on the subsequent GH secretory responses (deconvolution parameters) using a *α* = 0.10. Since this is a pilot study, the sample size is small, however, we also report effect sizes (omega‐squared [*ω* ^2^] and eta‐squared [*η* ^2^]) for the reader's determination of “significance.” When appropriate, post hoc analyses were conducted using a Bonferroni correction with an *α* < 0.05. Statistical computations were carried out using an SPSS Statistical software package (IBM; Armonk, NY).

## Results

### Participant characteristics

Table 1 presents descriptive characteristics of study participants. In the previous month prior to entry in this study, these women participated in approximately 412 ± 239 total minutes of structured physical activity (~91 ± 53 min/week) verifying that these women did not meet the ACSM physical activity recommendations. Additionally, the mean $\dot{V}O_{2\max}$ of these women was 29.4 ± 5.7 mL/kg/min, giving them a “very poor” classification based on ACSM guidelines (American College of Sports Medicine, 2014). All trials were completed during the (self‐reported) follicular phase of the menstrual cycle. Mean estradiol concentrations (pg·mL^−1^) between the three trials were similar (CON: 84.12 ± 72.8; MOD: 73.3 ± 45.7; HIE: 70.8 ± 44.7; *P* *=* 0.923).

**Table 1:** Participant descriptive characteristics

	Mean ± SD
Age (y)	22.6 ± 1.3
Height (cm)	159.2 ± 5.4
Weight (kg)	69.2 ± 7.6
BMI (kg/m^2^)	27.4 ± 3.1
Waist circumference (cm)	78.1 ± 5.0
Body fat (%)	39.2 ± 1.7
VO_2max_ (L/min)	2.03 ± 0.41
VO_2max_ (mL/kg/min)	29.43 ± 5.69
HR_max_ (bpm)	189.5 ± 10.5
Max power output (W)	183.8 ± 22.6

*n* = 5. SD = standard deviation; BMI, body mass index; VO_2max_, maximal oxygen consumption.

### Exercise responses

The mean power output on the cycle ergometer for the MOD trial was 80.6 ± 6.3 W, and steady‐state $\dot{V}O_{2}$ measured during the last 5‐min elicited a value equal to 68.2 ± 9.7% of $\dot{V}O_{2\max}$ (1.36 ± 0.11 L·min^−1^). The average heart rate during the MOD trial was 160 ± 17 bpm, which was equal to 83.7 ± 7.4% of HR_max_. The average peak lactate concentration during the MOD trial was 4.7 ± 0.9 mmol·L^−1^.

During the HIE trial, peak power output decreased from the first sprint (*P* = 0.004, *ω* ^2^ = 0.13). Compared to Sprint 1 (520.4 ± 95.6 W), peak power was significantly lower for Sprint 3 (460.8 ± 90.7 W, *P* = 0.039) and Sprint 4 (431.6 ± 67.0 W, *P* = 0.017. There was also a main effect for sprint number on mean power output (*P* < 0.01, *ω* ^2^ = 0.50). Compared to Sprint 1 (389.8 ± 45.7 W), mean power output was significantly lower for Sprint 2 (328.2 ± 38.7 W, *P* = 0.004), Sprint 3 (283.0 ± 43.5 W, *P* = 0.010), and Sprint 4 (298.8 ± 25.6 W, *P* = 0.012). The peak HR measured during the HIE trial was 183 ± 11 bpm, which was approximately 96% ± 4% of HR_max_. Both the peak HR (183 ± 11 bpm) and mean HR (180 ± 11 bpm) achieved during HIE were significantly greater than the mean HR during MOD (160 ± 17 bpm; *P* < 0.02). The peak lactate concentration during HIE was 11.1 ± 2.2 mmol·L^−1^ which was significantly higher than peak lactate concentrations during the MOD trial (4.7 ± 0.9 mmol·L^−1^; *P* = 0.007).

### Exercise lactate responses

**Figure 1:**
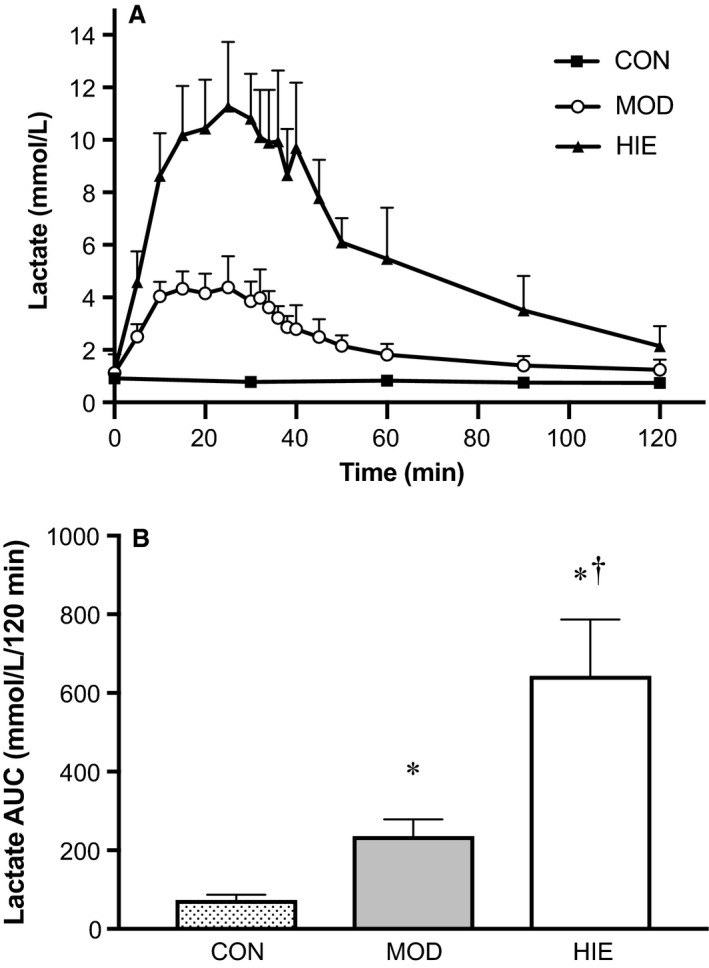
Lactate response. Lactate response to CON, MOD, and HIE during the first 120‐min of each trial. (A) HIE had the highest lactate response compared to MOD and CON (*P* < 0.01). (B) Mean lactate AUC by trial. *Compared to CON (*P* < 0.01), ^†^Compared to MOD (*P* = 0.025). Error bars represent ± SD. *n* = 5.

There was a significant main effect for exercise intensity on lactate response during the first 120 min of each trial (*P* < 0.01). There was also a significant interaction between exercise intensity and lactate response over time such that MOD resulted in a higher lactate response than CON (*P* = 0.008) and HIE resulted in a higher lactate response than MOD (*P* = 0.025) and CON (*P* = 0.004) (Fig. 1A). Calculated AUC (*n* = 5) demonstrated an intensity‐dependent increase in lactate response with exercise (*P* = 0.002) (Fig. 1B).

### Estimated exercising energy expenditure

Energy expenditure from both the MOD and HIE trial was estimated using power output data from the cycle ergometer. Mean energy expenditure from MOD (145.1 ± 11.2 kcal) was significantly higher than the HIE trial (97.4 ± 3.87 kcal; *P* = 0.001).

### Nonprotein respiratory quotient and nonesterified fatty acids

**Figure 2:**
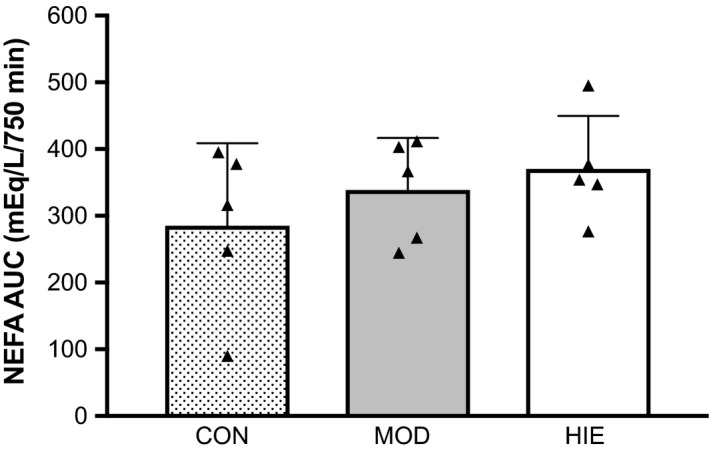
Calculated NEFA area under the curve (AUC) from 10‐min sampling over 750 min for CON, MOD, and HIE. There were no significant differences between trials. *n* = 5.

There was no difference in urinary nitrogen excretion (g N_2_·h^−1^) between the three trials (CON: 0.339 ± 0.07; MOD: 0.466 ± 0.10; HIE: 0.438 ± 0.11, *P* = 0.181). NPRQ 12 h after HIE (0.77 ± 0.02) was lower than NPRQ measured after MOD (0.80 ± 0.08) and CON (0.82 ± 0.02), but this difference was not significant (*P* = 0.087, *ω* ^2^ = 0.05). Nonesterified fatty acid concentrations measured over the 12.5 h time period were not significantly different between trials (*P* = 0.145, *η* ^2^ = 0.724). However, mean NEFA AUC was almost 30% greater after HIE compared to CON and ~10% greater compared to MOD (Fig. 2).

### Exercise growth hormone response

**Figure 3:**
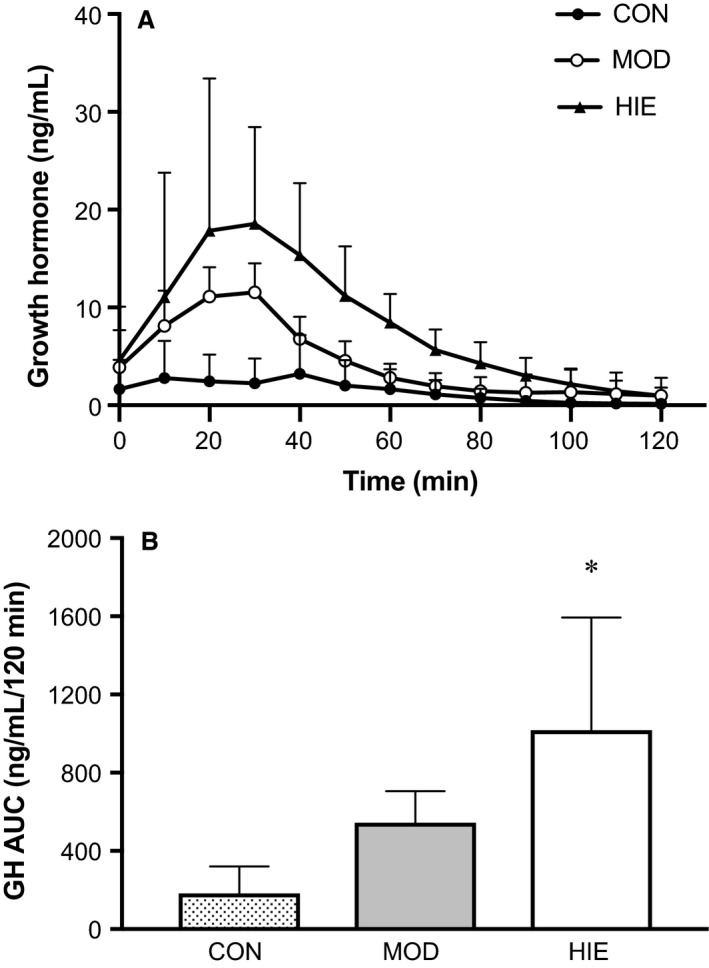
(A) Mean serum growth hormone response for 120‐min during CON, MOD, and HIE trials. Participants exercised from 0–30 min during the MOD and HIE trials. During the 0–30 min period (CON only) and the 30–120 min period (all trials) participants sat and watched TV or did homework. Error bars represent mean data ± standard deviation. (B) Calculated serum growth hormone area under the curve (AUC) from 10‐min sampling over 120 min for CON, MOD, and HIE. The calculated AUC over the 120‐min period was significantly greater after HIE compared to CON. *Compared to CON (*P* = 0.046). AUC is reported as mean ± SD. *n* = 5.

Figure 3 shows the mean (±SD) serum GH concentrations from blood sampled at 10‐min intervals over 2 h for the three conditions (CON, MOD, HIE). During the 120 min there was a significant effect for trial (*P* = 0.002, *ω* ^2^ = 0.21), such that GH concentration was higher during MOD compared to CON (*P* = 0.120) and significantly higher in HIE compared to CON (*v* = 0.042) (Fig. 3A). There was no significant difference in GH response between MOD and HIE (*P* = 0.641). There was also a significant effect for time on GH response (*P* = 0.011, *η* ^2^ = 0.78), however, due to large participant variability in GH response, comparisons between time points and trial did not reach statistical significance.

Calculated GH area under the curve values for 0–120 min are shown in Figure 3B. The 120‐min GH AUC response to exercise was significantly affected by intensity (*P* = 0.003, *ω* ^2^ = 0.485). Growth hormone AUC for CON (181.70 ± 138.99 ng·mL^−1^·120 min^−1^) was not significantly different from MOD (544.75 ± 160.68 ng·mL^−1^·120 min^−1^, *P* = 0.107), but was significantly lower than HIE (1018.23 ± 576.11 ng·mL^−1^·120 min^−1^, *P* = 0.046). There was no statistical difference in calculated GH AUC between MOD and HIE (*P* = 0.617).

### Growth hormone deconvolution analysis

**Figure 4:**
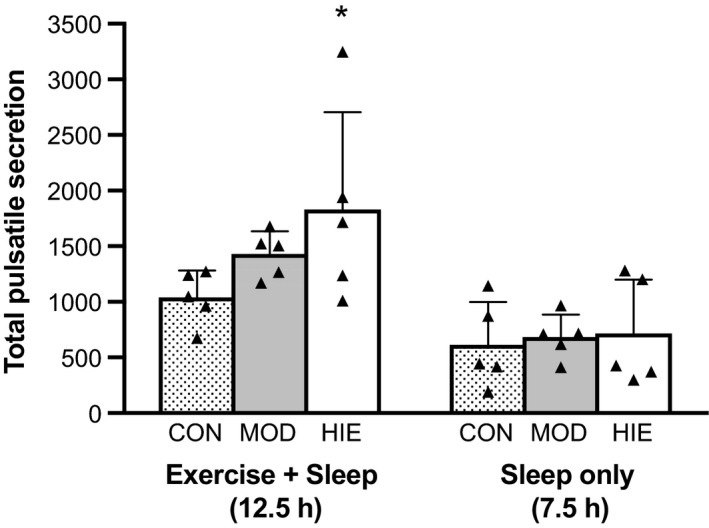
Estimated GH secretory rate for the total time frame (12.5 h) and during sleep only (7.5 h). Total pulsatile GH secretion (ng·mL ^−1^·12.5 h^−1^) was greater in HIE compared to CON for the total time (12.5 h) (*P* = 0.05). There were no differences in GH secretory rate (ng·mL ^−1^·7.5 h^−1^) during sleeping between the three trials. Values reported are mean ± SD. *n* = 5.

Deconvolution parameters (mean GH concentration [ng·mL^−1^], total pulsatile secretion rate [ng·mL^−1^·12.5 h^−1^], number of bursts, interval between bursts [min], mean burst height [ng], and mean area under bursts [ng·mL^−1^] are presented in Table 2. Due to large variability in individual responses, the deconvolution parameter data was log‐transformed. Using the log‐transformed data, total pulsatile secretion was significantly affected by trial (*P* = 0.081, *ω* ^2^ = 0.25) such that total GH pulsatile secretion during HIE was significantly elevated compared to CON (*P* = 0.050) (Fig. 4). Estimated pulsatile secretion during MOD was higher than CON (*P* = 0.083), but this did not reach significance; and there was no difference in pulsatile secretion between MOD and HIE (*P* = 0.518). Other deconvolution parameters measured were not different between the three trials.

**Table 2:** Deconvolution analysis parameters for GH concentrations across each trial

	CON	MOD	HIE
Mean GH	1.40 ± 0.31	1.93 ± 0.25	2.51 ± 1.36
Total secretion	1040.33 ± 241.96	1429.22 ± 205.94^a^	1831.20 ± 873.88^b^
Pulse #	10.0 ± 3.7	8.8 ± 1.3	9.2 ± 4.0
Interpulse interval	74.19 ± 46.78	80.00 ± 12.21	89.80 ± 42.14
Pulse height	0.61 ± 0.50	0.35 ± 0.09	0.45 ± 0.18
Pulse area	10.94 ± 7.17	9.42 ± 4.24	12.05 ± 4.89

Mean ± SD. *n* = 5. Units for mean GH (ng·mL^−1^); total secretion = total *pulsatile* secretion rate (ng·mL^−1^·12.5 h^−1^); pulse height (ng); interpulse interval (min); and pulse area (ng·mL^−1^·min^−1^).

a Compared to CON (*P* = 0.083).

b Different from CON (*P* = 0.050).

To remove the effect of exercise on total pulsatile secretion, data were analyzed for the time frame during which participants were in bed (2230–0600 h). There was no difference between trials in total pulsatile GH secretion rate for sleep only (*P* = 0.671, *ω* ^2^ = 0) (Fig. 4). There were also no differences in other deconvolution parameters between the three trials during sleep.

## Discussion

Exercise training is an effective nonpharmacological method to increase GH pulsatile secretion and circulating GH concentrations. A major action of GH is to stimulate free fatty acid mobilization from adipose tissue through pulsatile secretion, which leads to increased circulating fat available for oxidation (Cersosimo et al. 1996; Surya et al. 2009). The purpose of this pilot study was to determine if pulsatile GH secretion was increased by HIE compared to MOD and CON, and to measure the effect of an acute bout of exercise on substrate oxidation (NPRQ) across 12 h postexercise.

The major findings of this pilot study were as follows: (1) the GH response to HIE was augmented compared to CON, but not different from MOD; (2) total pulsatile GH secretion was increased in HIE compared to CON; and (3) overnight GH pulsatile secretion was not influenced by MOD or HIE, and substrate oxidation 12 h postexercise was not affected by intensity.

### GH response to exercise

The mechanism of action of GH on peripheral tissues is dependent upon its pulsatile delivery from the hypothalamus (Surya et al. 2009). Loss of GH secretion pulsatility is characteristic of the diminished GH concentrations observed with obesity (Veldhuis et al. 1995; Langendonk et al. 1999). Exercise training can increase plasma GH release (Felsing et al. 1992; Weltman et al. 1992, 2006; Kanaley et al. 1997; Pritzlaff et al. 1999; Pritzlaff‐Roy et al. 2002), the magnitude of which is intensity dependent (Pritzlaff et al. 1999; Pritzlaff‐Roy et al. 2002). GH calculated AUC (0–120 min) after HIE was significantly greater than the no exercise CON trial in this group of women. The moderate‐intensity bout was not significantly elevated from CON or different from HIE. Based on posteriori power analyses, a sample size of 10 women should yield a significant difference in GH secretion between CON and MOD; however, a sample size of 81 women would be needed to see a difference between MOD and HIE for these data, suggesting that both moderate and high‐intensity interval exercise secrete GH similarly.

Research consistently shows that exercise *above* the lactate threshold amplifies pulsatile release of GH, while exercise below the threshold does not (Luger et al. 1992; Weltman et al. 1992; Godfrey et al. 2003). In young, recreationally active men, pulsatile GH release increased linearly above baseline values with increasing exercise intensities both below and above the lactate threshold (Pritzlaff et al. 1999). In the current study, lactate threshold was not measured, but 3 of the 5 participants were at a $\dot{V}O_{2}$ above their estimated ventilatory threshold which is highly correlated with lactate threshold. Mean $\dot{V}O_{2}$ during the MOD trial was at approximately 68% $\dot{V}O_{2\max}$, and mean lactate concentration during the 30‐min bout was 3.9 ± 0.7 mmol/L, which is considered around the onset of blood lactate accumulation, further suggesting the work rate of these women was at an intensity high enough to promote GH secretion. Two of the three women that were at an exercise intensity above their ventilatory threshold had mean lactate concentrations during the moderate‐intensity session above 4.0 mmol/L, with one participant's value at 4.9 mmol/L. Thus, based on this data, it is reasonable to expect that the difference in GH secretion between MOD and HIE was similar for these women, and that the lack of difference between MOD and CON was due to small sample size.

One of the novel aspects of this study was the quantification of overnight pulsatile GH secretion following high‐intensity interval exercise, using deconvolution analysis. Few studies have assessed how exercise can influence pulsatile secretion and to what extent physical activity could potentially optimize GH secretory profile in a GH‐deficient population (e.g., obesity). The results of this study demonstrated that total pulsatile GH secretion was increased with HIE compared to no exercise. Moderate‐intensity exercise also increased total pulsatile secretion, but this increase was not statistically significant. The difference in GH pulsatile secretion was not dependent on total work done, as the HIE trial expended significantly fewer calories during exercise than MOD. Three 30‐min bouts of exercise at 70% $\dot{V}O_{2\max}$ separated throughout the day was sufficient to increase 24‐h GH secretion (Kanaley et al. 1997), and intermittent exercise (10 min bouts at 50% between LT and $\dot{V}O_{2\mathrm{peak}}$) scattered throughout the day similarly increased 24‐h GH secretion as a single 30‐min bout at the same intensity compared to control (Weltman et al. 2008). As expected, the magnitude of this response was attenuated in obese participants, but not different between males and females (Weltman et al. 2008).

While a few studies have examined the GH response to high‐intensity interval exercise, this is one of the first to measure the GH secretory response following exercise that also includes overnight secretion data. Nocturnal GH secretion accounts for approximately 85% of total daily GH output (Veldhuis et al. 2004), and it was therefore hypothesized that GH secretion would be augmented overnight following an acute bout of high‐intensity interval exercise compared to control. However, there was no difference in overnight GH secretion between the three trials. Kern et al. (1995) found a compensatory reduction in nighttime GH secretion with a single bout of moderate‐intensity long‐duration (4 h) exercise such that the 24 h GH concentration measured after exercise was not different from control. One of the major contributors to GH secretion at night is achievement of deep (slow‐wave) sleep (Van Cauter 2000). We did not measure sleep quality in the current study, nor did we provide a “familiarization” sleep session in our lab prior to participation. It is possible that participants in this study had “disturbed” sleep (e.g., they did not enter deep sleep), and thus the overnight secretory profile may not be as robust as the normal secretory profile would be.

### Regulation of substrate oxidation during and after exercise

For this study, measurement of substrate oxidation following exercise (by NPRQ) 12 h after a single bout of exercise demonstrated a nonsignificant intensity‐dependent increase in reliance on fat following MOD and HIE. Growth hormone stimulates release of FFA from adipocytes thereby increasing the circulation of FFA and subsequent oxidation in the liver (Johannsson 1999; Godfrey et al. 2003). A single bout of exercise can have a large influence on lipid metabolism through catecholamine secretion, insulin suppression, and GH release. It is well documented that moderate‐intensity exercise can increase fat oxidation both during and up to 24 h postexercise (Mulla et al. 2000; Magkos et al. 2009). Growth hormone is an important mediator of postexercise FFA mobilization, as inhibition of GH secretion by octreotide infusion suppressed postexercise lipolysis rates (Enevoldsen et al. 2007). Lactate is also known to promote GH secretion (Chwalbinska‐Moneta et al. 1996; Elias et al. 1997), and is released in an intensity‐dependent manner. The postexercise lipolytic rate following high‐intensity exercise has not been fully elucidated, but it stands to reason (given the aforementioned roles of catecholamines and growth hormone) that there would be an increase in fat oxidation and mobilization following high‐intensity exercise. Although we had a large effect size, there was no significant difference between the trials in NEFA concentrations. Examination of the mechanisms and physiological adaptations in fat metabolism following high intensity interval and sprint interval exercise is imperative for understanding the fat loss associated with this exercise.

### Summary

This is the first study to examine the influence of high intensity interval exercise on pulsatile growth hormone secretion that includes overnight GH measurements. We found that HIE increased total GH pulsatile secretion compared to control, but did not influence the overnight GH secretory pattern. The HIE also resulted in a significant increase in GH AUC measured up to 1.5 h postexercise. While MOD pulsatile GH secretion was higher compared to control, this difference was not significant. Further research into the effect that HIE has on GH secretion, and whether HIE training (e.g., 2+ weeks) would elicit a significant response requires additional investigation.

## Conflict of Interest

The authors have nothing to disclose.
